# Novel Scabies Mite Serpins Inhibit the Three Pathways of the Human Complement System

**DOI:** 10.1371/journal.pone.0040489

**Published:** 2012-07-11

**Authors:** Angela Mika, Simone L. Reynolds, Frida C. Mohlin, Charlene Willis, Pearl M. Swe, Darren A. Pickering, Vanja Halilovic, Lakshmi C. Wijeyewickrema, Robert N. Pike, Anna M. Blom, David J. Kemp, Katja Fischer

**Affiliations:** 1 Infectious Diseases Program, Biology Department, Queensland Institute of Medical Research, Brisbane, Queensland, Australia; 2 School of Veterinary Sciences, University of Queensland, Gatton, Queensland, Australia; 3 Department of Laboratory Medicine, Lund University, Malmö, Sweden; 4 Department of Biochemistry and Molecular Biology, Monash University, Melbourne, Victoria, Australia; University of Bern, Switzerland

## Abstract

Scabies is a parasitic infestation of the skin by the mite *Sarcoptes scabiei* that causes significant morbidity worldwide, in particular within socially disadvantaged populations. In order to identify mechanisms that enable the scabies mite to evade human immune defenses, we have studied molecules associated with proteolytic systems in the mite, including two novel scabies mite serine protease inhibitors (SMSs) of the serpin superfamily. Immunohistochemical studies revealed that within mite-infected human skin SMSB4 (54 kDa) and SMSB3 (47 kDa) were both localized in the mite gut and feces. Recombinant purified SMSB3 and SMSB4 did not inhibit mite serine and cysteine proteases, but did inhibit mammalian serine proteases, such as chymotrypsin, albeit inefficiently. Detailed functional analysis revealed that both serpins interfered with all three pathways of the human complement system at different stages of their activation. SMSB4 inhibited mostly the initial and progressing steps of the cascades, while SMSB3 showed the strongest effects at the C9 level in the terminal pathway. Additive effects of both serpins were shown at the C9 level in the lectin pathway. Both SMSs were able to interfere with complement factors without protease function. A range of binding assays showed direct binding between SMSB4 and seven complement proteins (C1, properdin, MBL, C4, C3, C6 and C8), while significant binding of SMSB3 occurred exclusively to complement factors without protease function (C4, C3, C8). Direct binding was observed between SMSB4 and the complement proteases C1s and C1r. However no complex formation was observed between either mite serpin and the complement serine proteases C1r, C1s, MASP-1, MASP-2 and MASP-3. No catalytic inhibition by either serpin was observed for any of these enzymes. In summary, the SMSs were acting at several levels mediating overall inhibition of the complement system and thus we propose that they may protect scabies mites from complement-mediated gut damage.

## Introduction

Scabies is a common transmissible parasitic skin infestation caused by the mite *Sarcoptes scabiei*. It spreads rapidly under crowded conditions [1] and is a major public health problem in socially disadvantaged communities, such as Australian Indigenous populations and in developing countries [2]. In some remote Australian Aboriginal communities over 70% of children present to clinics with scabies in their first two years of life, most of them acquiring their first infection at the age of one to two months [3]. Pruritic scabies lesions facilitate opportunistic secondary bacterial infections, particularly with Group A streptococci and *Staphylococcus aureus*, causing significant sequelae, *i.e.* cellulitis, septicemia and glomerulonephritis [4] and leading to the most extreme levels of Acute Rheumatic Fever and Rheumatic Heart Disease worldwide [5]. Pyoderma affects more than 111 million children globally, making it one of the three most common skin disorders in children along with scabies and tinea. Despite the alarming numbers, scabies remains a truly neglected infectious disease, which is in part due to the difficulty in obtaining sufficient numbers of mites for molecular studies. Emerging resistance to the currently available therapeutics against scabies, permethrin and ivermectin, emphasizes the need to identify novel drug targets [6].

In the epidermis the human complement system is an immediate host defense, which operates as a network of more than 35 plasma proteins. Activation of the system is triggered by immune complexes, carbohydrates or foreign surfaces and proceeds via one of three enzymatic cascades: the classical (CP), lectin (LP) and alternative (AP) pathways [7]. This leads to opsonisation and phagocytosis of the target, the release of anaphylatoxins, followed by the induction of inflammation and the formation of a membrane attack complex, which creates a pore in the target membrane causing cell lysis. Any successful human pathogen that is in contact with host plasma must have evolved a strategy to resist complement-mediated killing.

Burrowing scabies mites imbibe epidermal protein and plasma [8], [9], containing a multitude of diverse factors of the human innate immunity including complement proteins, such as C1q or C9, which were previously found in the mite gut [10], [11]. Thus, the mite has to be equipped with specific mechanisms to protect its gut membrane against the adverse effects of complement activation. Recently, we have characterized a multigene family of proteolytically active and inactive serine proteases [12], [13] that are present in the mite digestive system. Two of the catalytically inactive scabies mite proteases were found to interfere with the human complement activation by binding to complement proteins [10]. This resonates with previous observations that many pathogens avoid complement attack or complement-mediated inflammation by evading recognition via antibodies or MBL and/or by expressing complement inhibitors (reviewed in [14], [15]). Characterization of countermeasures against complement, evolved in hematophagous parasites such as trematodes, nematodes, leeches, mosquitoes, flies, triatomine bugs, ticks and mites, is a rapidly developing research field [16]. Schistosomes for example are exposed to complement in the bloodstream of their definitive hosts and employ several strategies to evade complement on multiple levels of the system [17]. A high turnover of surface antigens and a low intrinsic immunogenicity of the mambranocalyx were proposed as broad mechanisms [18]. Their effects are enhanced by multiple regulatory proteins, some of them acquired from the host, that slow down complement function by binding and inhibiting various complement factors (reviewed in [16]). At the other end of the broad spectrum of blood-feeding parasites, and most closely related to mites, are the acarid ticks [19]–[21]. Feeding times of ticks vary from less than 1 h to weeks, implying that they must possess mechanisms to overcome host defenses. The feeding success of a tick depends on its ability to locally suppress the host complement and coagulation systems, and a major adaptation to blood feeding was proposed to be a complex assortment of pharmacologically active saliva components that are released into the bite site [22]–[24]. To date transcriptomes of twelve tick species have been analysed [25]–[38], and as anticipated an increasing number of complement-inhibitory molecules are identified, concurrent with the observation of anti-complement activity in tick salivary gland extracts [39].

The complement inhibitor OmCI, a salivary protein released by the soft tick *Ornithodoros moubata,* [40]–[42] has been shown to directly bind to the central complement component C5 in a stable complex, thereby inhibiting cleavage of C5 to the anaphylatoxin C5a and the subunit of the membrane attack complex C5b. Further lipocalins with high sequence identity to OmCI have recently been described, namely TSGP2 and TSGP3 in *O. Savignyi,* and are proposed to target C5 by a common mechanism of action [43]. *Ixodes scapularis* and *I. ricinus* produce a family of homologous Isac-like proteins that behave as regulators primarily of the alternative pathway by preventing C3b and factor B inhibition [44]. Further homologous sequences have been described in several *Ixodes* species [28], [29], [45]–[48]. Tyson et al. investigated the specific mechanism by which one of these homologs, *I. scapularis* salivary protein 20 (Salp20), inhibits the alternative pathway [49], demonstrating that Salp20 directly binds to the regulatory complement protein properdin, thereby destabilising the complement C3 convertase (C3bBb) in the alternative complement pathway.

The classical, lectin and alternative complement pathways are activated and controlled by serine proteases. These proteases are endogenously regulated by human serpins [50], which usually function as irreversible inhibitors of serine and occasionally cysteine proteases [51]. Serpins control proteolytic pathways in animals, plants, fungi, bacteria and certain viruses [52] and regulate diverse physiological functions in vertebrates, such as the blood clotting, fibrinolysis, complement, hormone transport and inflammatory responses. The protein superfamily of serpins shows a highly conserved secondary structure [51], consisting of three major β-sheets, eight to nine α-helices, and an exposed reactive center loop (RCL). The RCL is located 30–40 residues from the C-terminus. After binding to the active site of a target protease similarly to a binding substrate, the RCL of an inhibitory serpin is specifically cleaved by the protease. This triggers a major conformational change in the inhibitor that disrupts the active site residues, thus facilitating the formation of an inactive, covalently linked serpin/protease-complex. The specificity and rate of interaction of inhibitory serpins can be regulated by interactions between proteases and cofactors with serpin exosites (reviewed in [51]). In addition, increasing numbers of non-inhibitory serpins have been described that use alternative binding sites and/or unknown mechanisms to serve, for example, as chaperones, hormone transporters, tumor suppressors and anti-angiogenic factors [52].

It has been previously hypothesized that serpins released with the tick saliva may interfere with host protease cascades involved in innate immunity [53], [54]. To date over 30 serpin-encoding tick cDNA sequences have been described [54]–[58], most of them contain signal sequences and are secreted in midgut and salivary glands [56]. Several studies on tick serpins provide evidence that these play important roles in the parasite – host interface and may be valuable targets for tick control. We describe here novel serpins that are secreted into the gut of scabies mites, which feed in the epidermal layers [59]. Skin protein [60] and serum components, such as host immunoglobulins [61] and complement components including C1q [10] and C9 [62], have been localized by immunohistology in the digestive tract of scabies mites infesting human skin. Hence, as seen in ticks, mites presumably have to overcome inflammation, complement activation and blood coagulation to successfully feed. Here we provide evidence that scabies mites may utilize two serpins to manipulate the human complement system.

## Methods

### Ethics Statement

All animals were handled in strict accordance with good animal practice as defined by the Australian code of practice for the care and use of animals for scientific purposes, and the National Health & Medical Research Council’s (NHMRC) Animal Code of Practice. Ethical approval for the production of polyclonal antibodies in mice was obtained from the Queensland Institute of Medical Research Animal Ethics Committee in compliance with the Code of practice, the NHMRC, and the Queensland government responsible authority [63]. Written informed consent was obtained from the crusted scabies patient for the collection of shed skin crusts, with the approval of the Human Research Ethics Committee of the Northern Territory Department of Health and Families and the Menzies School of Health Research.

### Cloning

A sequence analysis of cDNA libraries made from human scabies mites (*Sarcoptes scabiei;* [64]) revealed several EST clones with high similarities to serpins. The cDNA sequences of two scabies mite serpins (*SMSB3a*, cDNA clone Yv7088B02; GenBank accession no. JF317220, www.ncbi.nlm.nih.gov/GenBank; *SMSB4*, cDNA clone Yv5004A04, GenBank accession no. JF317222) were amplified using specific primers (5′ ACCGGTCGACGATTGTGATGAAGCTCAATTGGATC, 3′ ACCGCTGCAGCTAGAATCGATGCACTTCACCGATGAAC) for *SMSB3* and (5′ ACCGGTCGACAAACCTCAG CACCATTCTCAATCG, 3′ ACCGCTGCAGTCATGCAACAATCGCTTGCGATGCATAGGGGCG) for *SMSB4*, respectively, including the restriction sites *Sal*I and *Pst*I required for directional cloning into the pQE9 expression vector (Qiagen). The PCR products were digested at these sites, ligated into the vector, and transformed into *E.coli* XL1-blue (Stratagene; *SMSB3*) and BL21 (DE3) cells (Qiagen; *SMSB4*). Transformants were confirmed by DNA sequencing with BigDye 3.1 (Applied Biosystems) using T3 and T7 primers. Serpin names were given according to human homologues and their nomenclature.

### Heterologous Expression and Purification of SMSs

Recombinant proteins were expressed in *E. coli* and purified under denaturing conditions. Briefly, *E. coli* cells were cultivated in LB medium containing 100 µg/mL ampicillin at 37°C over night. After inoculation of 2YT medium containing the same concentration of ampicillin, the cells were grown at 37°C until an OD_600_ of 0.5–0.6 was reached and expression was induced by addition of 1 mM IPTG for 4 hours. Cells were collected by centrifugation at 4000×g at 4°C for 20 min, resuspended in 50 mM Tris, pH 8.0, 100 mM NaCl, 10 mM EDTA, 1 mM PMSF and lysed in the presence of 250 µg/ml lysozyme and 10 µg/ml DNAse at room temperature under continuous rotation. All of the following purification steps were performed at 4°C. After sonication of the spheroplasts by a Sonifier 250 (Branson), inclusion bodies were washed six times using 50 mM Tris, pH 8.0, 100 mM NaCl, 10 mM EDTA, 0.5% (v/v) Triton X-100 and retrieved by centrifugation (16,000×g for 20 min at 4°C), followed by solubilisation in 6 M guanidine hydrochloride, 50 mM Tris, pH 7.8, 1 mM DTT for 60 min. Proteins were further purified by nickel immobilized metal affinity chromatography (Qiagen): Solubilised protein was diluted 1∶1 with 6 M urea, 100 mM NaH_2_PO_4_, 10 mM Tris, pH 8.0, 5 mM imidazole, 150 mM NaCl, 1% (v/v) glycerol, 1 mM DTT and bound over night to a pre-equilibrated Ni-NTA matrix (Qiagen) on a rotating shaker. The matrix was loaded into a column (PolyPrep, BioRad) and washed with 10 column volumes of 6 M urea, 100 mM NaH_2_PO_4_, 10 mM Tris, pH 6.3, 5 mM imidazole, 150 mM NaCl, 1% (v/v) glycerol, 1 mM DTT. Bound proteins were eluted using 4 column volumes of 6 M urea, 100 mM NaH_2_PO_4_, 10 mM Tris, pH 8.0, 250 mM imidazole, 150 mM NaCl, 1% (v/v) glycerol and 1 mM DTT. Purified recombinant proteins were refolded for 3 hours in 300 mM L-arginine, 50 mM Tris, 50 mM NaCl and 5 mM DTT at pH 8.0 for SMSB3 and pH 10.5 for SMSB4 by drop wise addition using a Minipuls 3 pump (Gilson) at a flow rate of 0.03 ml/min under continuous stirring. Refolded proteins were concentrated using an Ultrasette Lab Tangential Flow Device (10 kDa cut off; PALL Life Sciences), followed by further concentration in centrifugal filters (Amicon Ultra, Millipore). Protein concentrations were determined by Bradford [65]. Molecular masses and purity were confirmed using SDS-PAGE analysis with silver and Coomassie blue R-250 staining. For all following assays, SMSs were buffer exchanged into the corresponding assay buffers using Zeba Desalt Spin columns (Pierce) directly before use. Chemical control experiments were performed for all assays to exclude buffer effects.

### Site-directed Mutagenesis

For functional analysis, the SMSs sequences were systematically mutated in the predicted hinge region [clones A **(**T368E, A372V) and B (T368P)] and RCL [clones C (G381H) and D (L382P)] of *SMSB3* using oligonucleotide-based site-directed mutagenesis. Equivalent *SMSB4* mutations in the hinge region [F (T422P)] and RCL [H **(**S435H)] were also introduced. The mutant molecules were expressed, purified and refolded as specified above for the wild type molecules.

### Complement Proteins

C1, C1r, C1s, C2, C3, C3b, C4, C4b, C5, C6, C7, C8, C9, factor B, factor D, and properdin were purchased from Complement Technology (Tyler, USA). Human MBL was purchased from Statens Serum Institute (Copenhagen, Denmark). C1q [66] and factor I [67] were purified from human plasma as described previously.

### Localization of SMSs and Human IgG by Immunohistochemistry

The production of antisera against scabies mite proteins, tissue preparation of human mite-infested skin samples and the immunolocalization of scabies mite proteins were accomplished as previously outlined [68]. Antibodies against purified recombinant SMSB3 and SMSB4 proteins were raised in C57BL/6 female mice. In order to confirm the specificity of the anti-sera against SMSB3 and SMSB4, purified SMS proteins were separated by SDS-PAGE and transferred onto an Immubilon-FL PVDF membrane (Millipore), blocked with Odyssey blocking buffer (*LI-COR* Biosciences) overnight at 4°C, and incubated for 1 h with 1:500 dilutions of primary antibody raised against the recombinant SMS proteins. Membranes were washed four times in PBS, followed by incubation for 1 h with a fluorescent secondary antibody (Goat anti mouse-IR dye λ_800 nm_) at 1:10,000 dilution. Proteins were visualized using an Odyssey Infrared Imaging System (*LI-COR* Biosciences).

Tissue samples of scabies mite-infected human skin were formalin-fixed overnight, paraffin embedded and cut into 4 µm serial sections. After deparaffinisation, the slides were washed with Milli Q water for 10 min followed by 15 min washes in PBS at pH 7.2. In order to reduce non-specific background staining, sections were blocked with casein (BACKGROUND Sniper, BioCare Medical) for 10 min, followed by overnight incubation at 4°C with 10% (v/v) goat serum in 1% (w/v) BSA in PBS. Endogenous peroxidase activity was blocked with 3% (v/v) H_2_O_2_ in blocking buffer. For the primary immunodetection of SMSB3 and SMSB4, the produced polyclonal mouse sera were incubated for 2 h at a dilution of 1:1000. Pre-bleeds from mice used for antibody production served as negative controls and were incubated on sections of the same series. Incubation with secondary anti-mouse-HRP antibodies (from DAKO EnVision system for SMSB4 and from BioCare Medical for SMSB3) was done for 40 min at room temperature. Anti-human IgG was used as a positive control to identify the mite gut on adjacent sections. These sections were incubated for 40 min at 4°C with an anti-human IgG-HRP polyclonal antibody (1:500; Sapphire Biosciences). All slides were washed in PBS and stained using the Vector NovaRed peroxidase substrate kit (Vector Laboratories). Counterstaining with hematoxylin was carried out as previously described. Slides were scanned using a Scan Scope XT microscope (Aperio Technologies) at 40× magnification.

### Enzyme Assays

Enzyme assays were performed in standard assay buffer (100 mM Tris-HCl, pH 8.2 10 mM CaCl_2_ and 0.05% (v/v) PEG 8000) for serine proteases, with commercially available methylcoumarin substrates (Sigma Aldrich) at 37°C in a final volume of 100 µl in 96-well plates. Enzyme assays with cysteine proteases were analyzed in 100 mM potassium phosphate, pH 6.0, 2.5 mM cysteine for Sar s 1c and 100 mM sodium acetate-HCl, pH 5.5, 1.5 mM EDTA and 1 mM dithiothreitol for cathepsin L. The house dust mite serine protease Der p 3 was kindly provided by Professor Wayne Thomas (Telethon Inst for Child Health Research, Perth, Australia) and the scabies mite cysteine protease Sar s 1c by Dr Masego Johnstone (QIMR, Brisbane, Australia). The recombinant human cathepsin L was kindly provided by Weiwen Dai (Monash University, Melbourne, Australia). Trypsin, Chymotrypsin, Elastase, Thrombin and Cathepsin L were purchased from Sigma Aldrich. Thrombin and cathepsin L were separately pre-incubated in their assay buffers at 37°C for 15 min before use. SMSs and active proteases were pre-incubated at 37°C for 10 min in the corresponding buffer, followed by addition of the substrate to start the enzyme reaction. Rates obtained in the absence of SMSs were defined as 100%. Fluorescence was continuously measured on a POLARstar Optima fluorescent microtiter plate reader (BMG Labtech, Melbourne, Australia) at 30 s intervals at excitation and emission wavelengths of 370 and 460 nm, respectively. Generally, the hydrolysis rate was recorded for 75 minutes and the linear part of the enzymatic reaction was taken to calculate the rates depending on the enzyme used.

SMS B3 was buffer exchanged into 100 mM Tris, pH 8.2, 10 mM CaCl_2_ and 100 µg/ml (10 µg total) of the serpin was incubated with 25 µg/ml (2.5 µg total) human leukocyte (neutrophil) elastase (Sigma Aldrich) at 37°C for 15 min. Subsequently, the reaction was inhibited by addition of the elastase-specific inhibitor *N*-methoxysuccinyl-Ala-Ala-Pro-Val-chloromethyl ketone (Sigma Aldrich) to a 10 µM final concentration and incubated at room temperature for 30 min. Control reactions containing no elastase were incubated under the same conditions with or without addition of the inhibitor. Aliquots (3 µg) of the produced samples were tested in duplicate assays for the deposition of complement component C9, as outlined below. Each experiment was independently repeated three times.

### Analysis of Protease/serpin Complexes

In order to analyze serpin/protease complexes, serpins were further purified by additional size exclusion chromatography to remove putative serpin aggregates. Refolded and concentrated protein samples were applied at 4°C to a Superdex 75 column (HR 10/30) using an ÄKTA HPLC system (GE Healthcare) and a 500 µl loop equilibrated with 4 column volumes of size exclusion buffer (50 mM Tris-HCl, pH 8.0, 150 mM NaCl, 50 mM arginine and 1 mM dithiothreitol). Proteins were eluted using 1.5 column volumes of size exclusion buffer at a flow rate of 0.5 ml/min with 0.5 ml fractions being collected. Fractions containing the serpins were identified by SDS-PAGE in conjunction with an assay examining the inhibitory effects of fractions on chymotrypsin activity. Serpin/protease complexes were analyzed using SDS-PAGE under non-reducing conditions and Western blotting after pre-incubation of the serpin with each protease in serpin buffer at 37°C for 15 min at serpin/protease ratios of 4:1, followed by 1 min incubation at 95°C and the addition of SDS-PAGE loading buffer.

For the formation of serpin/protease complexes between the purified, refolded SMSs and purified complement proteases (C1r, C1s), the molecules were incubated for 1 h at 37°C in GVB^2+^ buffer (5 mM veronal buffer, pH 7.35, 140 mM NaCl, 0.1% (w/v) gelatin, 1 mM MgCl_2_, 0.15 mM CaCl_2_) at serpin/protease ratios of 3:1 and boiled in non-reducing loading buffer at 95°C. The samples were separated by gradient SDS-PAGE (4-12%), transferred onto an Immubilon-FL PVDF membrane (Millipore), blocked with Odyssey blocking buffer (*LI-COR* Biosciences) overnight at 4°C, and incubated for 1 h with 1:2000 dilutions of primary antibody raised against the recombinant SMS proteins. Immunodetection was performed using fluorescent secondary antibody (Goat anti mouse-IR dye λ_800 nm_) at a dilution of 1:15000, followed by coomassie staining. Proteins were visualized using an Odyssey Infrared Imaging System (*LI-COR* Biosciences).

### Hemolytic Assays

To assess the activity of the classical pathway of complement, sheep erythrocytes (Swedish National Veterinary Institute) were washed three times using cold DGVB^2+^ buffer (2.5 mM veronal buffer, pH 7.35, 70 mM NaCl, 140 mM glucose, 0.1% (w/v) gelatin, 1 mM MgCl_2_, 0.15 mM CaCl_2_) and centrifugation (950×g, 4°C, 4 min), resuspended to a concentration of 10^9^ cells/ml and incubated, with gentle shaking, for 20 min at 37°C, with an equal volume of sensitizing antibodies (amboceptor, Dade Behring) diluted 1∶3000 in DGVB^2+^ buffer [10]. After two washes with DGVB^2+^ at 4°C, 8×10^7^ cells/ml were incubated for 1 h at 37°C with 0.125% normal human serum (NHS) in DGVB^2+^ buffer, in a total volume of 150 µl in 96-well plates under continuous shaking. Before incubation with erythrocytes, NHS was pre-incubated with various concentrations of SMSs or BSA, as a negative control, for 10 min at 37°C. NHS was prepared from blood of six to nine healthy volunteers after informed consent and following review by the local ethical board at Lund University or the Queensland Institute of Medical Research, respectively. Residual erythrocytes were separated by centrifugation (950×g, 4°C, 3 min) and the hemolytic activity, *i.e.* the amount of lysed erythrocytes, was determined by spectrophotometric measurement of the amount of released hemoglobin (λ = 405 nm). The lysis obtained in the absence of SMSs was defined as 100% hemolytic activity.

To assess the activity of the alternative pathway, rabbit erythrocytes (HeamoView Diagnostics, Pullenvale, Qld, Australia) were washed three times with Mg^2+^ EGTA buffer (2.5 mM veronal buffer (pH 7.3) containing 70 mM NaCl, 140 mM glucose, 0.1% gelatin, 7 mM MgCl_2_, 10 mM EGTA). Erythrocytes at a concentration of 6×10^7^ cells/ml were incubated for 1 h at 37°C with 1.6% NHS diluted in Mg^2+^ EGTA buffer in a total volume of 150 µl. Before incubation with erythrocytes, NHS was preincubated with various concentrations of SMSs or BSA, as a negative control, for 10 min at 37°. Residual erythrocytes were separated by centrifugation (950×g at 4°C for 3 min) and the hemolytic activity was determined spectrophotometrically as described above. The lysis obtained in the absence of SMSs was defined as 100% hemolytic activity.

### Complement Deposition Assays

Unless stated otherwise, all incubation steps were performed at room temperature, in a total volume of 50 µl, followed by extensive washing with 50 mM Tris-HCl, pH 8.0, 150 mM NaCl and 0.1% (v/v) Tween 20. Microtiter plates (96-well; Maxisorp, Nunc) were incubated overnight at 4°C with 50 mM sodium carbonate, pH 9.6 containing the following components: for the classical pathway, 2.5 µg/ml aggregated human IgG (Immuno); for the lectin pathway, 100 µg/ml mannan (Sigma-Aldrich); and for the alternative pathway, 20 µg/ml zymosan (Sigma-Aldrich). Coating with 1% (w/v) BSA was used as negative control. The wells were blocked with 250 µl of blocking solution (1% (w/v) BSA in PBS) for 2 h. To analyze the classical and lectin pathways, 1% and 2% NHS, respectively, were incubated in GVB^2+^ buffer (5 mM veronal buffer, pH 7.35, 140 mM NaCl, 0.1% (w/v) gelatin, 1 mM MgCl_2_, 0.15 mM CaCl_2_) for 20 min (for detection of the complement proteins C4b and C3b) or 60 min (for detection of C1q, MBL and C9) at 37°C. For the alternative pathway, 4% (v/v) NHS was incubated in Mg^2+^ EGTA buffer (2.1 mM veronal buffer, pH 7.35, 60 mM NaCl, 120 mM glucose, 0.1% (w/v) gelatin, 7 mM MgCl_2_, 10 mM EGTA) for 35 min for detection of C3b or for 1 h for detection of C9. Serum concentrations were chosen on the basis of initial titrations and represent conditions under which each assay is most sensitive to changes. The alternative pathway is known to require higher concentrations of serum to function properly. NHS was pre-incubated for 10 min at 37°C with various concentrations of SMSs or BSA, as a negative control, before addition to the microtiter plate. Complement activation was assessed by detection of deposited complement proteins using rabbit polyclonal antibodies against C1q, C4c, and C3d (Dako), or goat polyclonal antibodies against MBL (R&D Systems) and C9 (Complement Technology) diluted in blocking solution. After 1 h incubation with the primary antibody, HRP-conjugated secondary antibodies against rabbit or goat IgG (Dako) were diluted in blocking solution and added for 30 min (for C4b and C3b detection) or 60 min (for C1q, MBL, C9). Bound enzyme was detected using o-1,2-phenylenediamine dihydrochloride tablets (OPD, Dako) in presence of hydrogen peroxide and the absorbance was measured at a wavelength of 490 nm. The absorbance obtained in the absence of SMSs was defined as 100%.

### Direct Binding Assays

Unless stated otherwise, all incubation steps were performed in a total volume of 50 µl, followed by extensive washing, as described for the complement activation assays. Various purified complement proteins were coated onto microtiter plates as described above at a concentration of 10 µg/ml 1% (w/v) BSA was used as a negative control. The wells were blocked with 250 µl of blocking solution and incubated at room temperature for 2 h. SMS proteins (5-20 µg/ml) were diluted in binding buffer (50 mM HEPES, pH 7.4, 100 mM NaCl, 2 mM CaCl_2_) and allowed to bind for 4 h at 4°C. Specific mouse polyclonal antibodies against SMSs were then added to the wells, followed by an HRP-conjugated goat anti-mouse IgG antibody (Dako). All antibodies were diluted in blocking buffer at a ratio of 1:4000 for SMSs and 1:2000 for HRP-IgG and incubated at room temperature for 1 h. Bound HRP was detected as described above.

For NHS binding assays, microtiter plates were coated with 10 µg/ml recombinant purified SMSB3, SMSB4 and BSA as negative control in 50 mM sodium carbonate, pH 9.6 at 4°C over night. Coating with 1% (v/v) NHS was used as positive control. ELISA conditions were optimized for specific binding of the antibodies against individual complement proteins. After blocking as described above, wells were incubated with serial dilutions of NHS (0-100%) for 10, 20, 40 or 60 min at 37°C. Bound complement proteins were detected by incubation with primary antibodies against human complement factors for 1 h at room temperature (for immunodetection of C1r, MBL, C2, factor B, factor D and C8 at a dilution of 1:1000; for detection of C1s at 1:2500; for detection of C1q, C3d, C4c, C6 and properdin at 1:4000). To further confirm that complement serine proteases were not interfering with the ELISA, i.e. digesting the antibodies, assays for the detection of the heat-sensitive proteins factor B and C2 were additionally performed with and without heat-inactivation (15 min at 56°C) after NHS binding. Depending on antibody specificity and signal intensity, rabbit anti-goat/HRP and goat anti-rabbit/HRP secondary antibodies were applied for 30 min or 1 h (at dilutions of 1∶1000-1∶5000) in blocking buffer. Bound HRP was detected at 490 nm as described above.

### Statistical Analysis

All experiments were carried out at least three times in duplicate, unless stated otherwise. Statistical significance was determined using one-way ANOVA (Origin 5.0 Professional software). Values of *p<*0.05 were considered significant.

### Ethics Statement

All animals were handled in strict accordance with good animal practice as defined by the Australian code of practice for the care and use of animals for scientific purposes, and the National Health & Medical Research Council’s (NHMRC) Animal Code of Practice. The Queensland Institute of Medical Research Animal Ethics Committee approved the production of polyclonal antibodies in mice for this study, in compliance with the Code of practice, the NHMRC, and the Queensland government responsible authority. Written informed consent was obtained from the crusted scabies patient for the collection of shed skin crusts. The protocol was approved by the Human Research Ethics Committee of the Northern Territory Department of Health and Families and the Menzies School of Health Research.

## Results

### Sequence Analysis and Recombinant Expression of SMSs

**Figure 1:**
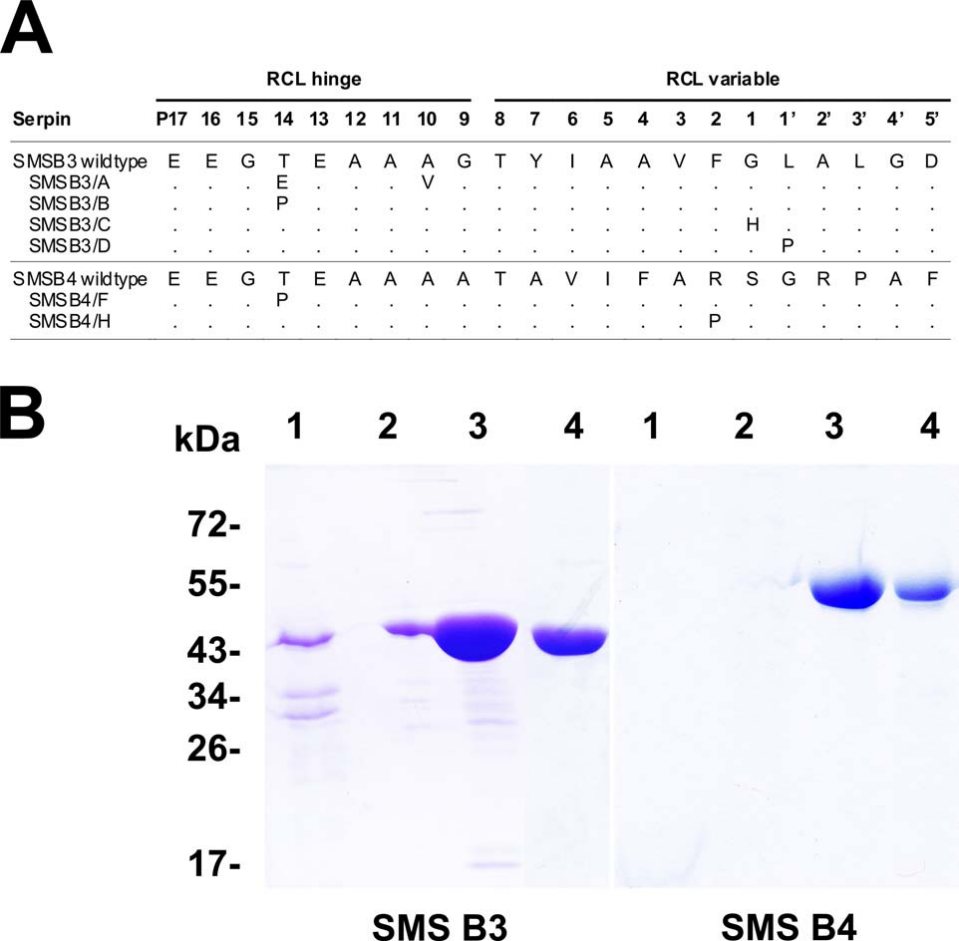
Sequence details and recombinant expression of SMSs and mutants. (A) Shown are the sequence changes in the SMS mutants compared to wild type sequences. Numbering scheme according to [82]. Point mutations were introduced in the two segments of the RCL domain: i) the distal (P9-P5’) variable region that resembles the protease substrate and is attacked by a target protease, and ii) the proximal (P15-P9) hinge region that is well conserved and inserts into β-sheet A during inactivation of a protease in inhibitory serpins. **(B)** Purification and refolding of SMSB3 and SMSB4. Shown are immobilized nickel affinity chromatography fractions after separation by SDS-PAGE and Coomassie staining. Both SMSs revealed the expected bands of 47 kDa and 54 kDa, respectively. 1, loaded sample; 2, wash; 3, elution with 250 mM imidazole, 4, active serpin after refolding.

Two novel cDNA sequences coding for the scabies mite serpins SMSB3 and SMSB4 were amplified by PCR from scabies mite cDNA libraries. The corresponding full-length amino acid sequences consisted of 427 and 498 residues, respectively, and had predicted signal sequences indicating that they are secretory proteins. The theoretical molecular masses and isoelectric points were 46.5 kDa and 5.61 for SMSB3 and 53.6 kDa and 5.92 for SMSB4, respectively. Both SMSs showed sequence similarities to human clade B serpins and to serpins from other arthropods such as ticks, lice and house dust mites (47-62%). Both scabies mite sequences revealed the typical domains and highly conserved residues of the serpin superfamily. SMSB4 contained an additional 103 amino acid residue long N-terminal elongation, which is different from human homologues and other mammalian serpins. In order to analyze the inhibitory mechanism of the scabies mite serpins, six mutants were created introducing point mutations within the RCL hinge or RCL variable regions (Fig. 1A).

All proteins investigated in this study were expressed in *E. coli* with N-terminal hexaHis-tags and purified from thoroughly washed inclusion bodies under denaturing conditions by affinity chromatography. Subsequent refolding in presence of L-arginine and dithiothreitol yielded an average of 6 mg SMSB3 and 160 µg SMSB4 per liter of induced culture, *i.e.* 20–30% of the purified proteins were refolded into soluble proteins. The purified proteins exhibited the expected molecular masses of 47 and 54 kDa, respectively (Fig. 1B). The six mutated serpins were successfully produced following the same procedure and showed identical molecular masses and purity compared to the corresponding wild type SMSs on SDS-PAGE (data not shown).

### SMSs are Localized in the Mite Digestive System and Excreted into the Epidermis

**Figure 2:**
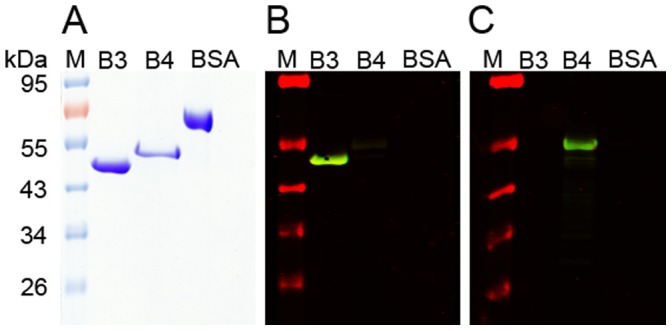
Specificity of mouse antisera raised against recombinant SMSB3 and SMSB4. The purified SMS proteins and BSA (used as a non-related control) were electrophoresed on SDS-PAGE and stained with Coomassie blue R-250 (A) and then immunoblotted with the anti-sera raised against SMSB3 (B) and SMSB4 (C), confirming the specificity of the antibodies and lack of cross-reaction with BSA.

**Figure 3:**
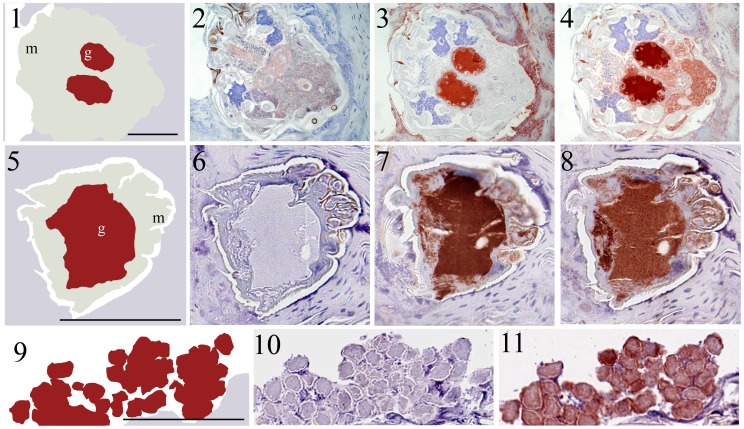
Immunolocalization of SMSs and IgG in scabies mite-infested human skin. Schematic diagrams **1** and **5** outline the features visible in serial histological sections through a scabies mite in infested human skin. Red staining indicates binding of antibody to protein. Section **2** and **6** probed with pre-immune mouse serum as a negative control remained unstained, while equivalent regions were detected in section **4** and **8** when probed with anti-human IgG, a marker for mite gut [9], and in section **3** and **7** when probed with antibodies against SMSB4 and SMSB3, respectively. Schematic diagrams outline the features visible in serial histological sections through human epidermis containing scabies mite feces **9**. Section **10** probed with pre-immune mouse serum as a negative control remained unstained, while equivalent regions were detected in section **11** when probed with anti-SMSB3 and in section **12** with anti-IgG. Staining of feces was also seen in equivalent experiments performed with antibodies against SMSB4 (not shown). Immunohistological co-localization of SMSs and host IgG indicated that both serpins are localized in the mite gut and mite feces within the burrows. b, burrow; f, feces; g, gut; m, mite. Scale bars (100 µM) indicate the magnification level.

The specificity of the anti-sera raised against SMSB3 and SMSB4 was confirmed by Western analysis (Fig. 2). The presence of both SMSs in the scabies mite gut was demonstrated by probing serial sections of human mite-infested skin with SMS-specific antibodies in comparison to staining with human IgG, which for the purpose of this experiment served as a positive control for gut localization (Fig. 3). Human IgG was localized to the gut (Fig. 3, section 4 and 8) as previously documented [9], [68].This confirmed that, in the adjacent serial section (Fig. 3, section 3 and 7), labelling by anti-serpin Abs was indeed in the gut. The polyclonal antibody sera used for the immunolocalization studies were raised in mice against *E. coli*-derived SMSB3 and SMSB4 and reacted specifically with each protein in western blot analysis (Fig. 2). In addition to their location in the digestive tract of the mite, the SMSs were localized to external acellular masses, known as fecal pellets. This is exemplarily shown for SMSB3 (Fig. 3, section 11). In all cases, the sections probed with the pre-immune serum as negative control only stained with the counterstain, clearly excluding any unwanted background staining (Fig. 3, sections 2, 6 and 10). From these results we conclude that both SMSs are located to the digestive tract of the scabies mite and are excreted into the epidermis with the feces of the parasite. Consequently, the serpins are highly likely to be involved in functions within the gut of the mite, but equally may interact with host proteins present in the epidermis after excretion in the feces.

### SMSs Inhibit Mammalian Proteases and Form Serpin/protease Complexes

**Figure 4:**
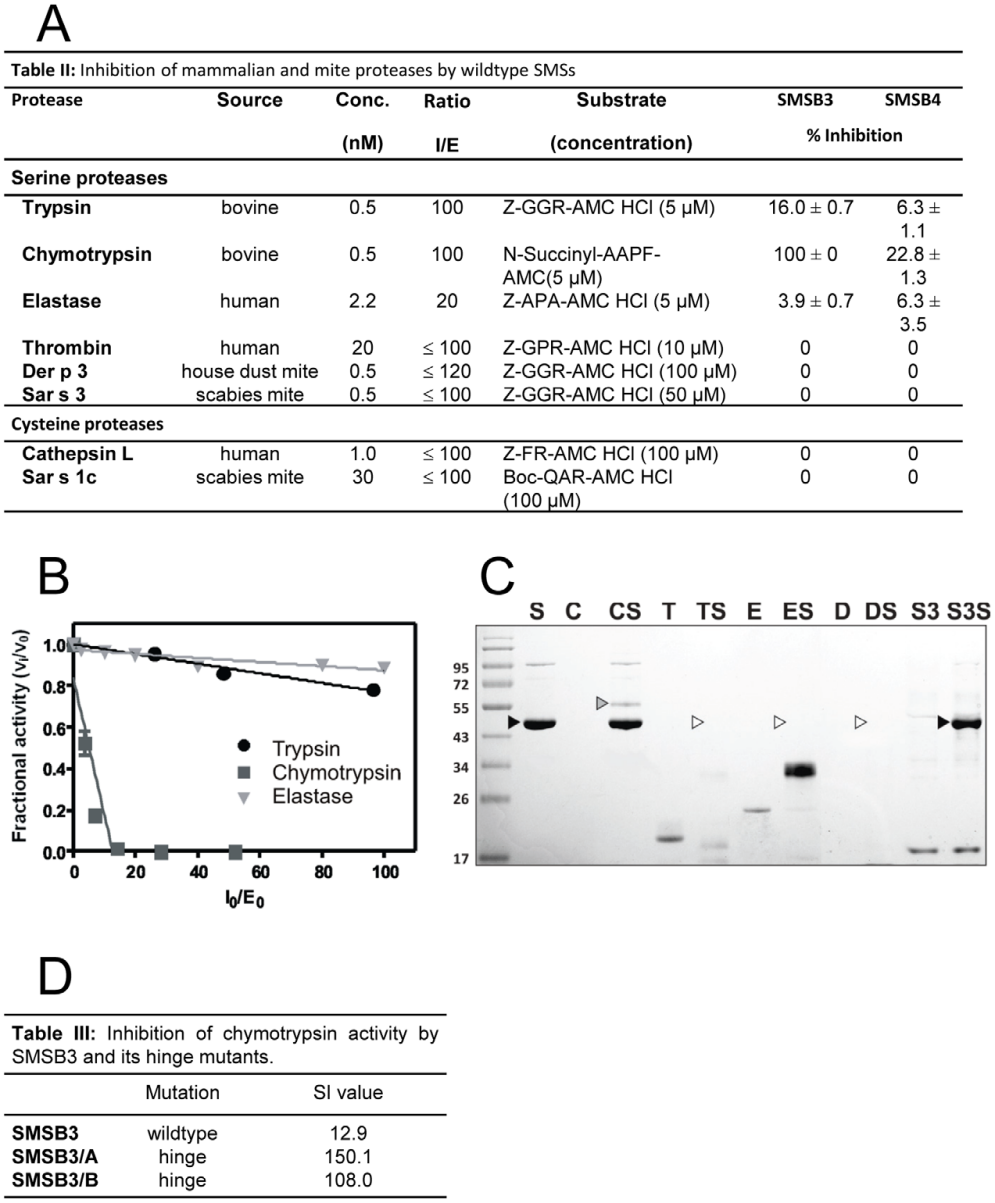
SMSs inhibit mammalian proteases and form serpin/protease complexes. (**A)** Inhibitory profile of mammalian and mite proteases by wild type SMSs (overview). Proteases were pre-incubated with different concentrations of SMSB3 or SMSB4 at 37°C for 10 min in the appropriate reaction buffer. The reaction was started by addition of the corresponding fluorescent aminomethyl-coumarin substrate and enzyme inhibition was measured as change in fluorescence in comparison with controls. Shown are means ± SD (n = 3). **(B)** Stoichiometry of inhibition for SMSB3. The inhibitory activity was assessed by measuring residual enzyme activities of trypsin, chymotrypsin and leucocyte elastase. Enzymes were incubated with a 2-120:1 molar excess of SMSB3 at 37°C for 10 min in the appropriate reaction buffer. Residual enzyme activity was measured in triplicate (SD ≤5%) by adding the appropriate aminomethyl-coumarin peptide substrate and determining the reaction velocity as change in fluorescence. The fractional activity was the ratio of the velocity of inhibited enzyme (v_i_) over the velocity of uninhibited control (v_o_). The initial inhibitor/enzyme ratios ([I_0_]/[E_0_]) represent the molar excess of serpin over enzyme. (**C)** Complex formation between SMSB3 and serine proteases. Serpin/protease complexes were analyzed by SDS-PAGE under non-reducing conditions after pre-incubation of SMSB3 (black arrow) with each protease at 37°C for 15 min at serpin/protease ratios of 4∶1. Covalent complex formation was found with chymotrypsin (grey arrow), while SMSB3 was degraded by trypsin, elastase and Der p 3 (white arrows). C, chymotrypsin; D, house dust mite Der p 3; E, neutrophil elastase; S, SMSB3; S3, scabies mite Sar s 3; T, trypsin. (**D)** Effects of SMSB3 and two mutants on chymotrypsin activity.

Enzyme activity assays in the presence and absence of both wild type SMSs revealed that SMSB3 and SMSB4 are active and preferentially inhibit mammalian serine proteases, such as trypsin, chymotrypsin and human leukocyte elastase (Fig. 4A). In contrast, the scabies mite serine protease Sar s 3, its house dust mite homolog Der p 3 and the cysteine proteases Sar s 1c and human cathepsin L were not inhibited by either SMS. Values for the stoichiometry of inhibition (SI) of the three mammalian proteases by SMSB3 (Fig. 4B) were elucidated using linear regression to determine the inhibitor/enzyme ratio ([I]_0_/[E]_0_) resulting in the complete inhibition of the enzyme. While trypsin and elastase inhibition by SMSB3 and SMSB4 were inefficient, the SI value calculated for SMSB3 inhibition of chymotrypsin was 12.9, indicating that this serpin inhibited chymotrypsin-like proteases most effectively.

Serpin/protease complexes formed by the classical mechanism involving RCL cleavage and formation of an acyl-enzyme intermediate are heat and SDS-stable and hence can be visualized after SDS-PAGE. Such analysis of SMSB3/protease complexes confirmed that the inhibition of chymotrypsin was due to the formation of a covalent bond between the enzyme and the inhibitor (Fig. 4C). In contrast, the slight inhibition of trypsin and elastase activity in presence of SMSB3 apparently occurred due to the serpin acting as an alternative substrate for the enzymes, based on the degradation products visible on SDS-PAGE analysis. Scabies mite serine protease Sar s 3 did not affect the scabies mite serpin (Fig. 4C). Western blotting of the SDS-PAGE gels after complex formation and immunodetection with antibodies against SMSB3 confirmed that the high molecular mass complex band formed contained SMSB3 (data not shown).

SMSB3/A and SMSB3/B showed strongly impaired inhibitory activity against chymotrypsin in comparison with the wild type serpin (Fig. 4D). These mutants contain point mutations at P14 in the RCL-associated hinge region of SMSB3 (Fig. 1A), indicating that this region, which is integral to the conformational change occurring during the serpin mechanism, was indeed involved in the inhibition of this protease.

### SMSsB3 and SMSB4 do not Inhibit Blood Coagulation

We tested the functionality of the intrinsic and the extrinsic coagulation pathways, involving multiple serine proteases, in the presence of the two recombinant mite serine protease inhibitors SMSB3 and SMSB4. Two standardized tests, the Activated Partial Thromboplastin Time (APTT) and the Prothrombin Time (PT) were assessed. Both SMSs had no effect on either pathway when tested at concentrations of 10, 100 and 200 µg/ml (215 nM, 2.1 µM and 4.2 µM for SMSB3 and 186 nM, 1.8 µM and 3.6 µM for SMSB4) (Fig. S1).

### SMSs inhibit the hemolytic activity of human serum

**Figure 5:**
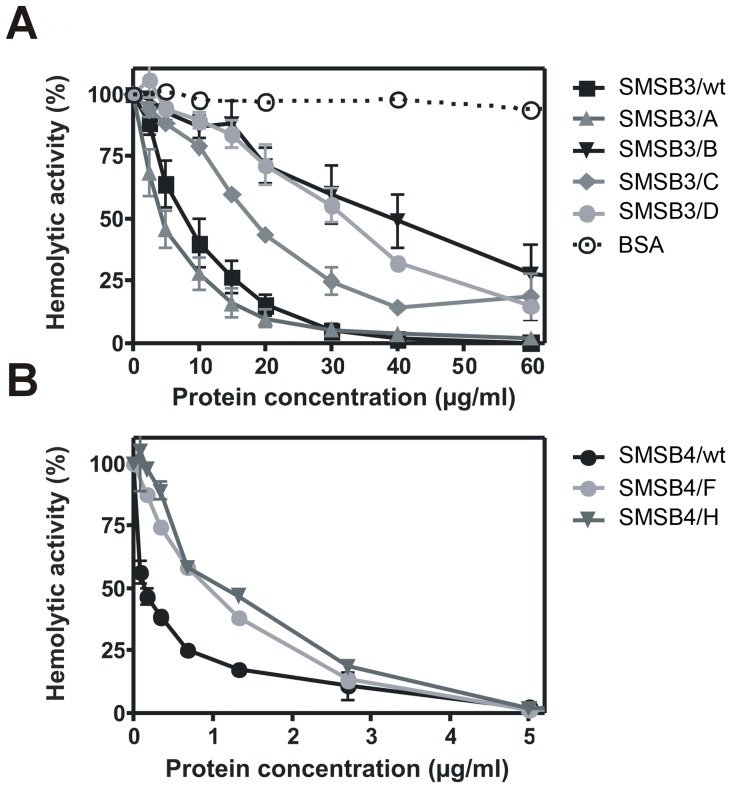
SMSs inhibit the hemolytic activity of human serum. Sheep erythrocytes opsonized with antibodies were incubated with 0.125% NHS to visualize the activity of the classical pathway of the human complement system. Serum was pre-incubated for 10 min at 37°C with various concentrations (2.5–60 µg/ml, i.e. 54–1300 nM) of SMSB3 and its four mutants (**A**), SMSB4 and its two mutants (0.1–5 µg/ml, i.e. 2–93 nM) (**B**) and BSA as a negative control. After 1 h of incubation of NHS with erythrocytes at 37°C, the degree of complement-mediated lysis was estimated by measurement of released hemoglobin. The lysis obtained in the absence of SMSs was defined as 100% hemolytic activity. An average of three independent experiments performed in duplicate is presented with bars indicating SEM.

A detailed functional analysis was performed to investigate the anti-complement properties of the scabies mite serpins. Both SMSs inhibited human complement in a hemolytic assay measuring the activation of the classical pathway, while the six mutants (Fig. 1A) showed distinct changes in inhibition (Fig. 5). SMSB3 and SMSB4 reached total inhibition of complement-mediated lysis of sheep erythrocytes at protein concentrations of 40 µg/ml (860 nM) and 5 µg/ml (93 nM), respectively.

While SMSB3/A, a molecule containing a double point mutation in the hinge region (Fig. 1A), was as efficient as wild type SMSB3 in its ability to inhibit complement, the mutants SMSB3/C, SMSB3/D and SMSB3/B were significantly impaired in their ability to inhibit the classical pathway (Fig. 5A). The unchanged activity of SMSB3/A in comparison with the wild type serpin revealed that the hinge region required for the serpin inhibitory mechanism is not important for the inhibition of human complement by SMSB3, in contrast to the inhibition of chymotrypsin. The serpins containing proline mutations in the RCL or hinge region (SMSB3/D and SMSB3/B) required 4-fold and 5-fold higher protein concentrations, respectively, to cause 50% inhibition of the hemolytic activity in comparison with the wild type serpin (Fig. 5A). Similarly, 50% inhibition was reached using 7 and 8-fold higher protein concentrations of the SMSB4 RCL and hinge mutants in comparison to wild type SMSB4 (Fig. 5B).

Experiments addressing the alternative pathway in hemolytic assays showed 40% inhibition in the presence of 100 µg/ml of wild type SMSB3, compared to no inhibition in the BSA control (data not shown).

### SMSs Inhibit All Three Complement Pathways

**Figure 6:**
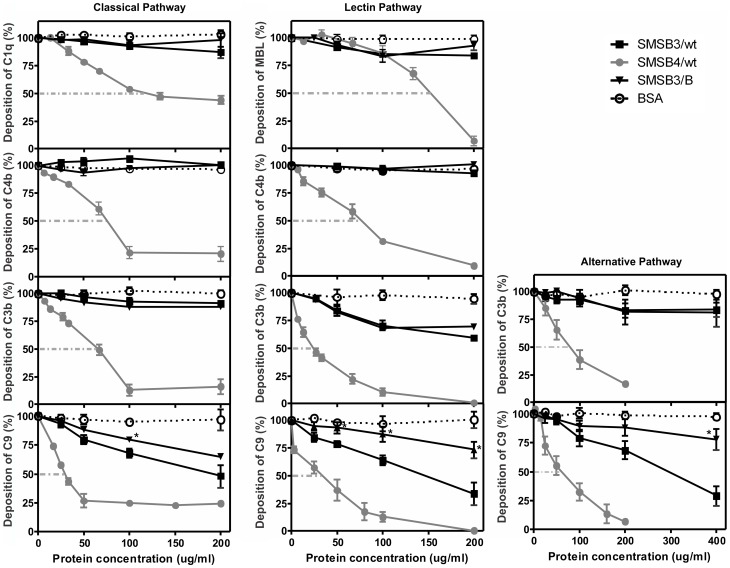
SMSs inhibit the classical, lectin and alternative pathway. Complement deposition assays were performed on microtiter plates coated with aggregated IgG (classical pathway), mannan (lectin pathway) and zymosan (alternative pathway) in presence or absence of scabies mite serpins, respectively. Shown are means ± SEM of n = 4 independent experiments, each performed in duplicate. The three pathways were measured using 1% (CP), 2% (LP) and 4% (AP) NHS. Assays were performed at inhibitor protein concentrations of 25–400 µg/ml (0.5–8.6 µM) for SMSB3/wt and SMSB3/B and 10–200 µg/ml (0.2–3.7 µM) for SMSB4/wt. Significant differences between wild type SMSB3 and the hinge mutant SMSB3/B (*; p<0.05).

Further analysis characterizing the deposition of human complement factors at different levels within the three activated complement pathways, revealed that SMSB3 and SMSB4 inhibited all three pathways by interfering with several steps of the cascades (Fig. 6). In an ELISA-based functional assay, aggregated IgG, mannan or zymosan were used as activators of the classical, lectin and alternative pathways, respectively, leading to the deposition of the pathway-specific complement factors. After addition of human serum, pre-incubated with SMSs, changes in the deposition of complement proteins were detected using antibodies specific to individual complement components, allowing assessment of the level at which inhibition of the complement pathway occurred.

SMSB4 inhibited at the C1q level, and stronger inhibition was seen at the C4b and C9 levels, as indicated by the significantly lower serpin concentrations needed to reach 50% inhibition of the classical pathway (Fig. 6). In the lectin pathway the presence of SMSB4 resulted in a significant decrease in the deposition of MBL, and a significantly greater decrease in the deposition of C4b, C3b and C9 (Fig. 6). Strong inhibition occurred at the C3b level in the alternative pathway (Fig. 6), but no significant change in complement inhibition was found in the terminal pathway (C9). In contrast, SMSB3 exerted its strongest inhibition at the C9 level of all three complement pathways, and only weak inhibition at the level of C3b in the lectin pathway. Higher concentrations of both SMSs were needed to inhibit the alternative pathway, which is most likely due to differences in the serum concentrations used to assess the three pathways (Fig. 6). In summary, SMSB4 seems to be a more potent inhibitor of all three pathways of the human complement system than SMSB3. While both serpins acted on several steps within the three pathways, SMSB4 interfered predominantly with the initial and progressing levels of the cascades, while SMSB3 exerted its strongest effects at the C9 level in all three pathways.

Due to the low protein purification yields of some of the SMS mutants, SMSB3/B was chosen as a model molecule to investigate the inhibitory mechanism against complement in deposition assays. SMSB3/B, containing the point mutation T368P in the RCL hinge region of the serpin, showed significantly different effects at the terminal complement pathway level (C9) in comparison with wild type SMSB3 (Fig. 6).

### SMSs Act Accumulatively on Deposition of Complement Component C9

**Figure 7:**
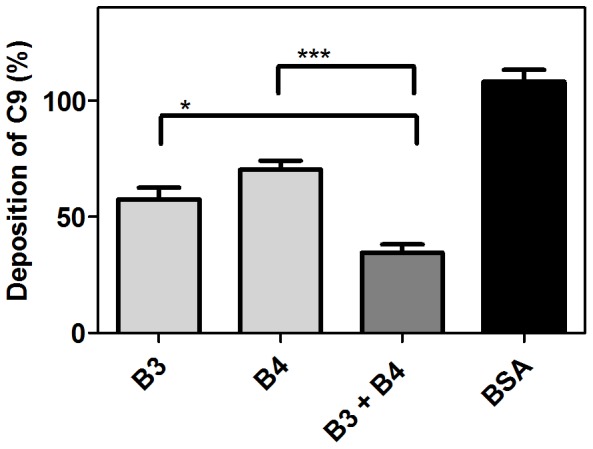
Additive effects of SMS B3 and SMSB4. Mannan was immobilized on microtiter plates and allowed to activate 4% NHS containing 5 µg/ml of either serpin (110 nM SMSB3 or 93 nM SMSB4) alone or mixed together. As a negative control BSA was used at a concentration of 10 µg/ml. After 20 min of incubation the plates were washed and the deposited C9 was detected with specific antibodies. An average of three independent experiments is presented with bars indicating means ± SEM. *p<0.05, ***p<0.001 by t test (GraphPad Prism).

Since both serpins are present simultaneously in the mite gut we addressed how they acted on complement when present together. The lectin pathway was recently shown to play a role in scabies mite biology and a novel peritrophin localized in the mite gut was identified as a potential binding target of MBL [11]. As both serpins showed inhibition of this pathway we have assessed their collective effects in inhibiting the deposition of C9. A single dose of each serpin at a concentration of 5 µg/ml (110 nM SMSB3 or 93 nM SMSB4) yielded a 43% and 30% decrease of C9 deposition respectively, while the combination of both serpins at the same concentrations decreased C9 deposition by 66%, thereby indicating additive effects (Fig. 7).

### Digestion of SMSB3 by Human Neutrophil Elastase has no Effect on C9 Deposition in the Lectin Pathway

**Figure 8:**
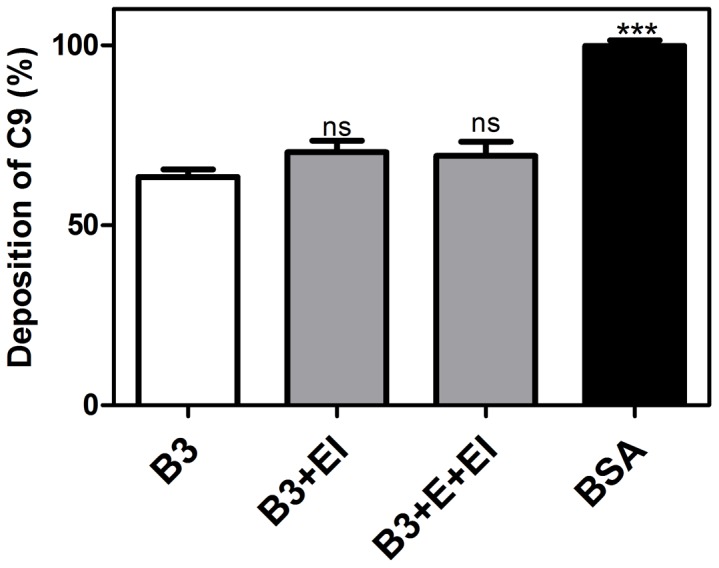
Digestion of SMSB3 by human neutrophil elastase has no effect on C9 deposition. SMSB3 was cleaved with human leukocyte elastase, following which the enzyme was inhibited by addition of *N*-methoxysuccinyl-Ala-Ala-Pro-Val-chloromethyl ketone. Control reactions without addition of elastase (E) and elastase inhibitor *N*-methoxysuccinyl-Ala-Ala-Pro-Val-chloromethyl ketone were preformed under the same conditions. Mannan was immobilized on microtiter plates and allowed to activate 4% NHS containing 50 µg/ml SMSB3. As a negative control, BSA was used at a concentration of 10 µg/ml. After a 20 min incubation the plates were washed and the deposited C9 was detected with specific antibodies. An average of three independent experiments is presented with bars indicating means ± SEM. *p<0.05, ***p<0.001 by t test (GraphPad Prism).

Elastase from recruited neutrophils may be present in the scabies mite infested skin during opportunistic bacterial infection. As the cleavage of SMSB3 by human neutrophil elastase could potentially be relevant for disease progression, we investigated whether cleavage of this serpin by elastase could alter the interaction with complement. For the lectin pathway, an increased concentration of serpin SMSB3 (50 µg/ml) was required to decrease C9 deposition by 40% under these conditions, but there was no significant difference in the effect on the deposition of complement component C9 between cleaved and whole SMSB3 (Fig. 8).

### SMSs Bind Directly to Several Complement Factors

**Figure 9:**
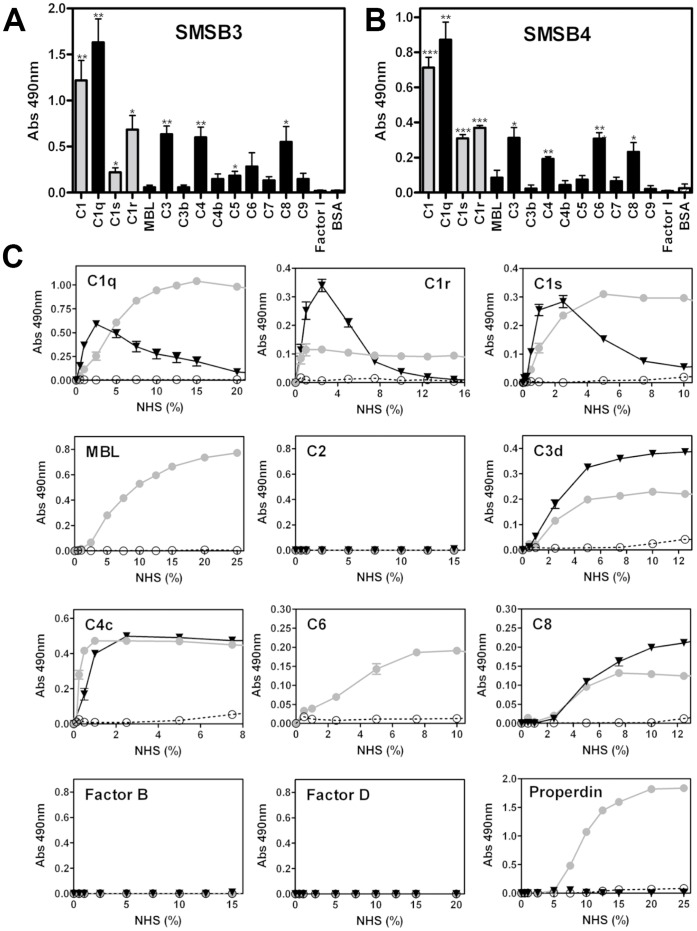
Direct binding of SMSs to various complement proteins. Microtiter plates were coated with various purified human complement proteins or BSA as a negative control and incubated with 20 µg/ml SMSB3 (**A**) or SMSB4 (**B**). Bound serpins were detected using specific polyclonal antibodies against SMSB3 and SMSB4. Shown are means ± SEM of n = 3 independent experiments. The statistical significance of differences between BSA and the rest of the data groups was estimated using one-way ANOVA. *, p<0.05; **, p<0.01; ***, p<0.001. Grey, serine proteases; black, other complement factors. **C** Direct binding of complement factors from NHS to SMSs. Increasing concentrations of NHS were added to wells coated with SMSB3 (▾, black), SMSB4 (•, grey) or BSA as a negative control (○, white) and bound complement factors were detected by specific antibodies. Shown are means ± SEM of n = 3 independent experiments measured in duplicates. NHS was tested from 0–100%. Positive controls were used for complement proteins, where no binding to the SMSs was detectable confirming strong immunodetection of the complement factor on 1% NHS coating.

SMSB3 and SMSB4 revealed significant binding to a range of purified human complement factors, and showed differences in specificity and affinity (Fig. 9A and B). While both SMSs bound strongly to the purified C1 complex and its single components, several other serine proteases and plasma complement proteins of the three cascades reacted with both SMSs, suggesting alternative binding sites. The mutant SMSB3/B showed the same binding pattern as the active serpin (data not shown), indicating that binding can still occur, but conformational changes of the serpin molecule required for complement inhibition may be impaired. Interestingly, direct binding of both SMSs to complement factors C3 and C4 occurred only with the native forms, *i.e.* C3 and C4 were bound, but not the cleaved factors C3b and C4b.

To confirm the direct binding of SMSs to human complement factors under more physiological conditions, additional binding assays were performed using NHS and immunodetection of the bound proteins (Fig. 9C). Typical hyperbolic curves were observed with increasing NHS concentrations. Direct binding was found between SMSB4 and the serine proteases C1r and C1s, respectively (Fig. 9C). In addition, SMSB4 bound to seven other complement factors (C1q, MBL, properdin, C3, C4, C6, and C8; Fig. 9C). The mechanism by which the serpin binds these non-protease factors is unknown, but the data confirm the inhibition of the complement pathways at many levels, including the start of the cascades (Fig. 6).

Low amounts of non-specific binding occurred between SMSB3 and the C1 complement components at low NHS concentrations (Fig. 9C). These were not seen at higher concentrations. Experiments using NHS confirmed the observed inhibitory effects on the terminal pathway by revealing typical binding curves for three complement factors (C3, C4, C8; Fig. 9C) indicating high affinity binding for these proteins. None of these factors have a serine protease function.

### Interaction of Scabies Serpins with Human Complement Enzymes

C1r or C1s, purified from human plasma, and recombinant MASP-1, MASP-2 or MASP-3 were incubated with SMSB3, SMSB4 or human C1-inhibitor and subsequently the samples were separated by SDS-PAGE (Fig. S2). As expected, C1s and C1r formed a higher MW band after incubation with human C1-inhibitor, formed by the serpin interacting with the catalytic domain of these enzymes. However, the formation of the higher MW band was not seen with either of the scabies mite serpins when incubated with any of the complement proteases tested, indicating that the serpins were not able to form stable, covalently bound complexes with these proteases.

To investigate the interaction of the mite serpins with human MASP-1 and MASP-2, further increasing concentrations of purified recombinant MASP-1 or MASP-2 were incubated with SMB3 and SMB4 and analysed by SDS-PAGE. SMS B3 was cleaved by both MASP-1 and MASP-2, with higher concentrations of MASP-1 required to demonstrate the effect (Fig. S3). SMS B4 was unaffected by the enzymes and neither serpin could be shown to form an SDS-stable complex with the enzymes, indicating that they did not form the classical covalent enzyme-serpin complex. In order to investigate whether the serpins were able to inhibit the enzymes, increasing concentrations of the serpins were added to constant amounts of the enzymes and residual activity was monitored using the substrate, Leu-Gly-Arg-NHMec. No inhibition of enzyme activity was seen for either serpin with any of the enzymes (data not shown).

## Discussion

In this study two novel SMSs were biochemically characterized and functionally analyzed. Both SMSs showed some typical properties of the serpin superfamily [51]. Enzyme assays demonstrating the inhibitory profile of both scabies mite serpins showed that the recombinant purified proteins were active serpins (Fig. 4), able to inhibit mammalian proteases. SMSB3 inhibited chymotrypsin (Fig. 4) by formation of covalent serpin/protease complexes, presumably executing the classical serpin mechanism. This was further supported by the finding that SMSB3 mutants A and B with the hinge sequence altered at position 14 did not inhibit chymotrypsin (Fig. 4). It has been established previously that a mutation of the P14 residue of the hinge sequence in serpins generally blocks loop insertion and partially or completely abolishes serine proteinase inhibitory activity [69], as the flexibility of the hinge is centered on the presence of an uncharged P14 residue [70].

Immunohistochemistry studies showed that serpins localized in the scabies mite gut (Fig. 3). Host serum is ingested into the mite gut [9], [68], and the presence of complement factors, such as C1q and C9, has been demonstrated [10], [71]. It has been suggested that blood-feeding arthropods produce serpins that protect the gut from host circulatory proteases [52]. In line with this research, the discovery of complement-inhibitory serpins in the gut of scabies mites may add new aspects to our understanding of the scabies mite-host relationship. Apart from the gut, both serpin molecules were found in the mite feces, as shown for SMSB3 in Fig. 3, for example. As house dust mite allergens are known to be highly active proteases after excretion [72], [73] scabies mite gut molecules may be equally functional externally after being released into the upper epidermis, as well as internally within the gut.

SMSB4 exhibited effects as early as the initial and progressing steps of the complement cascades, while SMSB3 showed the strongest effects on the terminal pathway. Differences between protein concentrations needed in hemolytic and complement deposition assays were based on differences in NHS concentrations and other assay conditions. C1q is a pattern recognition molecule, which activates the classical pathway and assembles into a complex with its associated serine proteases, C1s and C1r [7]. The strong binding of SMSB4 to the components of the C1 complex (C1q, C1s and C1r; Figs. 9B and C) corresponds with the inhibitory effects at the C1q level demonstrated in deposition assays (Fig. 6). In contrast, the presumably non-specific binding between C1q and SMSB3 at low NHS concentrations (Fig. 9C) did not inhibit complement deposition (Fig. 6). Proteins binding to the complement factor C1q without affecting its function, or causing activation of complement instead, have been previously described [74]–[76].

C3 acts as the central molecule of the alternative pathway. Its hydrolyzed form is activated by the serine proteases B and D generating an initial C3 convertase, which cleaves C3 into C3a and C3b [7]. Properdin recognizes pathogen- or damage-associated molecular patterns and is involved in the initiation of the alternative pathway and the stabilization of AP convertases [77]. Thus, the binding of C3 and properdin by SMSB4 correlates with the strong inhibition at the C3b level in the alternative pathway (Fig. 6).

In contrast, SMSB3 only targeted non-serine proteases in human serum (Fig. 9C) and exhibited its strongest effect at the C9 level of the three pathways (Fig. 6) by interaction with C8, which takes part in the assembly of the terminal membrane attack complex along with the factors C5b, C6, C7 and C9.

An increasing number of serpins have been found to be non-inhibitory towards proteases and show alternative binding mechanisms [51], [52], [78]–[80]. The changes in anti-complement activity of the mutants, in comparison with the wild type SMSs (Fig. 5), and the binding of both SMSs to complement factors without protease function (Fig. 9), suggested that possibly one or several exosites might be used to inhibit these proteins. While the ability of both SMSB3 hinge mutants A and B to inhibit bovine chymotrypsin in enzyme assays was impaired, most likely by inhibition of the serpin mechanism, the anti-complement activity of the hinge mutant SMSB3/A was not changed. In contrast, the hinge mutants SMSB3/B and SMSB4/F showed impaired anti-complement activity (Fig. 5), suggesting that domains outside the hinge region may have to be structurally changed to impair the anti-complement function of the serpins. In the SMSB3/B mutant, a proline residue was inserted into the hinge region. Proline insertions often disrupt the secondary structure and thus misfolding of the protein can occur and/or conformational changes necessary for binding or inhibitory mechanisms elsewhere in the molecule can be affected. No complex formation was seen with either of the scabies mite serpins when incubated with any of the complement proteases C1s, C1r, MASP-1, MASP-2 and MASP-3 tested, indicating that the mite serpins were not able to form stable, covalently bound complexes with these proteases. Thus, it appears that the effect of the mite serpins is not due to classical serpin inhibition of the serine proteases of the complement cascade. Based on the data presented here, it can be hypothesized that both the complement proteases tested and the non-proteolytic complement factors likely bind to an exosite of the SMSs or that the SMSs bind to exosites on the proteases, causing these proteins to be sterically inhibited from completing further binding interactions required for complement activation. However, the potential alternative binding mechanisms of the serpins to the complement proteases and factors have yet to be elucidated.

SMSs may act in concert with each other (Fig. 7) and with the catalytically inactive scabies mite serine proteases [10], which also inhibit human complement. It appears that the scabies mite has evolved a multitude of mechanisms to ensure human complement inactivation at all pathway levels, similarly to the broad range of anti-complement factors evolved by other pathogens [17], [81]. We hypothesize that the compilation of mite complement inhibitors accumulates to high anti-complement activities in the confined space of the gut and epidermal burrows. Importantly, while complement factors are ingested by mites infesting human skin, MAC formation is not detected in the gut [71], suggesting that this anti-complement machinery may be very efficient *in vivo*. While prevention of gut lysis seems to be the obvious role of intestinal mite complement inhibitors, they may also act external to the mite and their presence in the epidermis may possibly have further consequences for the host. We have previously proposed that mite excretory proteins may effectively enhance the survival of scabies associated pathogenic bacteria by interfering locally with host complement [10]. The serpins described here may take part in this role. Further studies are needed to elucidate this hypothetical model of host, parasite and bacteria interactions in order to further the development of novel preventative and therapeutic strategies.

## Supporting Information

Figure S1 **Scabies mite serpins SMSB3 and SMSB4 do not interfere with the coagulation pathway.** The functionality of the intrinsic and the extrinsic coagulation pathways in the presence of the two recombinant serine protease inhibitors, SMSB3 and SMSB4, was assessed by measuring the Activated Partial Thromboplastin Time (APTT) and the Prothrombin Time (PT). The dotted boxes represent reference ranges for clotting times of healthy donors.
(PDF)

Figure S2 **Interaction of scabies mite serpins with human complement enzymes.** C1r (hC1r) or C1s (hC1s) purified from human plasma and recombinant MASP-1 (M1), MASP-2 (M2) or MASP-3 (M3) were incubated with SMSB3 (B3), SMSB4 (B4) or human C1-inhibitor (hC1i) for 1 h at room temperature. Samples were electrophoresed on 10% SDS-PAGE as indicated in the labels above each lane. The positions of the catalytic chains of the enzymes are indicated [e.g. C1r (c)]. The positions of the complexes between hC1r and hC1s and hC1i are also indicated.
(PDF)

Figure S3 **Interaction of scabies mite serpins with human MASP-1 and MASP-2.** Increasing concentrations of purified recombinant MASP-1 (M1) or MASP-2 (M2) were incubated with SMSB3 (B3) and SMSB4 (B4) for 1 h at room temperature. Samples were separated on 10% SDS-PAGE as indicated in the labels above each lane shown. Molecular weight markers (Precision Plus Protein™ Dual Colour Standard, BIO RAD) are shown in the first lane at the left of each SDS-PAGE gel.
(PDF)
