# Supplementary material for: Novel Scabies Mite Serpins Inhibit the Three Pathways of the Human Complement System
Source: PLoS One. 2012 Jul 11;7(7):e40489. doi: 10.1371/journal.pone.0040489 (PMC3394726; doi:10.1371/journal.pone.0040489)
Supplement: Figure S1 — Scabies mite serpins SMSB3 and SMSB4 do not interfere with the coagulation pathway. The functionality of the intrinsic and the extrinsic coagulation pathways in the presence of the two recombinant serine protease inhibitors, SMSB3 and SMSB4, was assessed by measuring the Activated Partial Thromboplastin Time (APTT) and the Prothrombin Time (PT). The dotted boxes represent reference ranges for clotting times of healthy donors. (PDF) [file pone.0040489.s001.pdf]

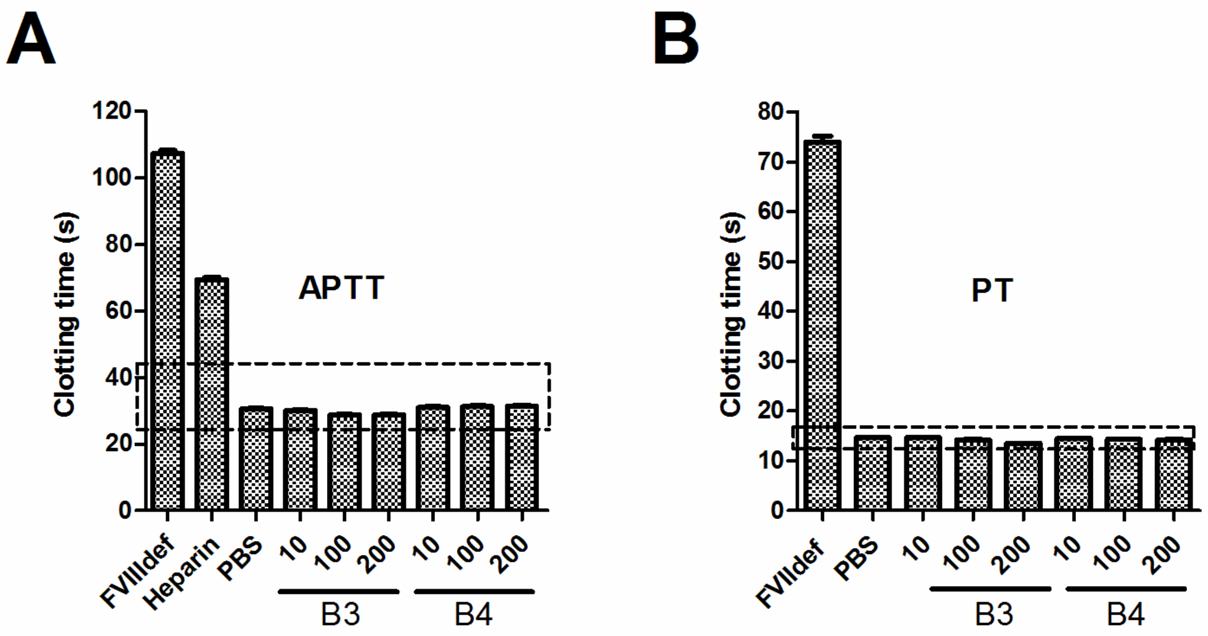

Figure S1: Scabies mite serpins SMSB3 and SMSB4 do not interfere with the coagulation pathway. The functionality of the intrinsic and the extrinsic coagulation pathways in the presence of the two recombinant serine protease inhibitors, SMSB3 and SMSB4, was assessed by measuring the Activated Partial Thromboplastin Time (APTT) and the Prothrombin Time (PT). The dotted boxes represent reference ranges for clotting times of healthy donors.

The SMSs were transferred into PBS buffer using micro desalt spin columns (Pierce). A blood sample in citrate vacutainers (Greiner) was taken from a healthy volunteer after informed consent and following review by the local ethical board at the Queensland Institute of Medical Research. The sample was centrifuged at 4200×g, 24°C, for 10 min to separate the plasma from blood cells. For each blood coagulation experiment, 50 µL protein was added to 800 µL plasma. An equivalent volume of PBS to the sample was used as a negative control. The addition of heparin (Sigma Aldrich) at a final concentration of 0.3 IU/ml and a factor 8 deficient plasma sample (Helena Laboratories, Texas, USA) served as positive controls for an effect on the APPT, while a factor 7 deficient plasma sample (Diagnostic Grifols, S.A., Spain) served as positive control for an effect on the PT. Tubes were incubated for 10 min at 37°C in a water bath and measured using a Sta-R coagulometer (Diagnostica Stago, Asnières, France). The kits TriniCLOT APTT HS (Trinity Biotech) and Thromborel S (Siemens) were used for the determination of the APTT and the PT, respectively. Both SMSs were tested twice, in duplicate and at three concentrations (10 µg/ml, 100 µg/ml and 200 µg/ml).

Discussion: The SMSs had no effect on either pathway when tested at these concentrations, achieving the same clotting time as PBS buffer, while prolongation of the Activated Partial Thromboplastin Time (APTT) was seen when factor 8 deficient plasma or healthy plasma supplemented with heparin at a final concentration of 0.3 IU/ml were tested and prolongation of the Prothrombin Time (PT) was seen when factor 7 deficient plasma was tested.
