# Supplementary material for: Novel Scabies Mite Serpins Inhibit the Three Pathways of the Human Complement System
Source: PLoS One. 2012 Jul 11;7(7):e40489. doi: 10.1371/journal.pone.0040489 (PMC3394726; doi:10.1371/journal.pone.0040489)
Supplement: Figure S2 — Interaction of scabies mite serpins with human complement enzymes. C1r (hC1r) or C1s (hC1s) purified from human plasma and recombinant MASP-1 (M1), MASP-2 (M2) or MASP-3 (M3) were incubated with SMSB3 (B3), SMSB4 (B4) or human C1-inhibitor (hC1i) for 1 h at room temperature. Samples were electrophoresed on 10% SDS-PAGE as indicated in the labels above each lane. The positions of the catalytic chains of the enzymes are indicated [e.g. C1r (c)]. The positions of the complexes between hC1r and hC1s and hC1i are also indicated. (PDF) [file pone.0040489.s002.pdf]

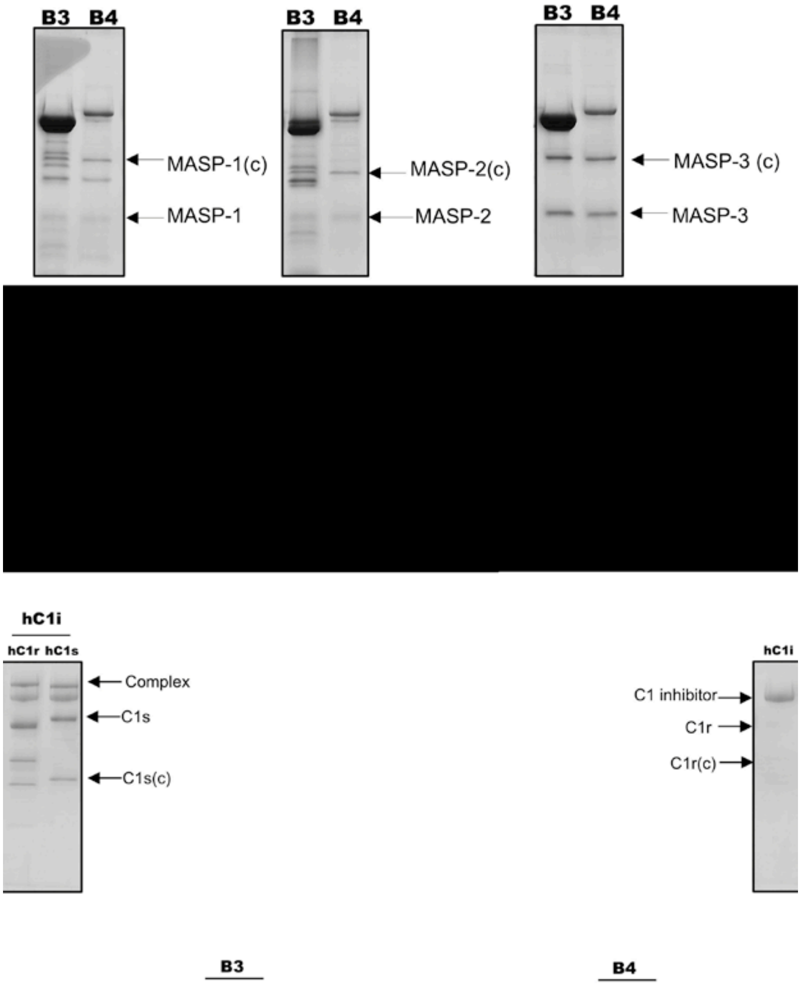

Figure S2: Interaction of scabies mite serpins with human complement enzymes.
C1r (hC1r) or C1s (hC1s) purified from human plasma and recombinant MASP-1 (M1), MASP-2 (M2) or MASP-3 (M3) were incubated with SMSB3 (B3), SMSB4 (B4) or human C1-inhibitor (hC1i) for 1 h at room temperature. Samples were electrophoresed on 10% SDS-PAGE as indicated in the labels above each lane. The positions of the catalytic chains of the enzymes are indicated [e.g. C1r (c)]. The positions of the complexes between hC1r and hC1s and hC1i are also indicated.

Discussion: As was seen with human C1-inhibitor, human C1s and C1r showed a higher MW band formed by the serpin interacting with the catalytic domain of these enzymes. The formation of the higher MW band was not seen with either of the scabies mite serpins when incubated with any of the proteases tested, indicating that the serpins were not able to form stable, covalently bound complexes with these proteases. It does appear that MASP-2 may be cleaving the serpin SMSB3 in its RCL to generate a lower MW form.
