# Supplementary material for: Novel Scabies Mite Serpins Inhibit the Three Pathways of the Human Complement System
Source: PLoS One. 2012 Jul 11;7(7):e40489. doi: 10.1371/journal.pone.0040489 (PMC3394726; doi:10.1371/journal.pone.0040489)
Supplement: Figure S3 — Interaction of scabies mite serpins with human MASP-1 and MASP-2. Increasing concentrations of purified recombinant MASP-1 (M1) or MASP-2 (M2) were incubated with SMSB3 (B3) and SMSB4 (B4) for 1 h at room temperature. Samples were separated on 10% SDS-PAGE as indicated in the labels above each lane shown. Molecular weight markers (Precision Plus Protein™ Dual Colour Standard, BIO RAD) are shown in the first lane at the left of each SDS-PAGE gel. (PDF) [file pone.0040489.s003.pdf]

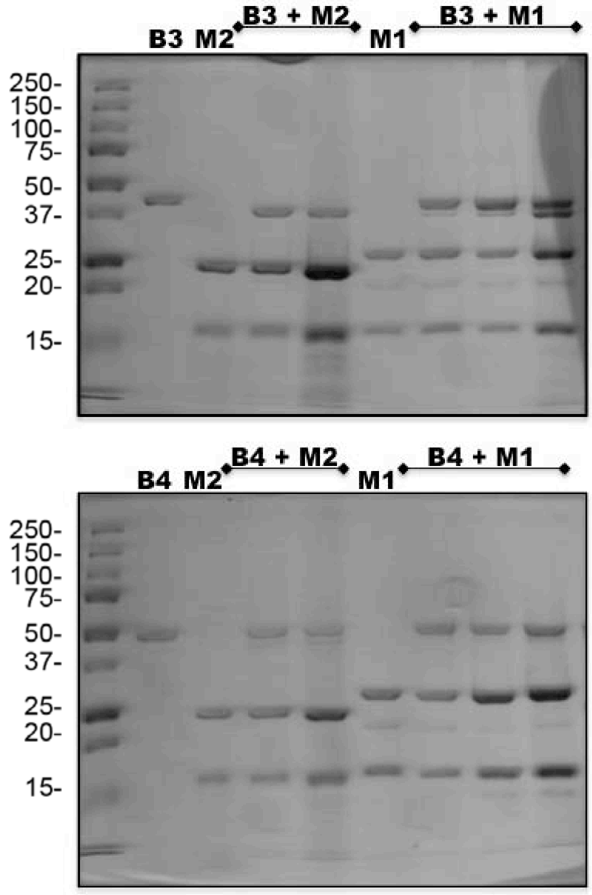

Figure S3:Interaction of scabies mite serpins with human MASP-1 and MASP-2.
Increasing concentrations of purified recombinant MASP-1 (M1) or MASP-2 (M2) were incubated with SMSB3 (B3) and SMSB4 (B4) for 1 h at room temperature. Samples were separated on 10% SDS-PAGE as indicated in the labels above each lane shown. Molecular weight markers (Precision Plus Protein™ Dual Colour Standard, BIO RAD) are shown in the first lane at the left of each SDS-PAGE gel.

Discussion: The SMSB3 was cleaved by both MASP-1 and MASP-2, with higher concentrations of MASP-1 required to demonstrate the effect. SMSB4 was unaffected by the enzymes and neither serpin could be shown to form an SDS-stable complex with the enzymes, indicating that they did not form the classical covalent enzyme-serpin complex.

In order to investigate whether the serpins were able to inhibit the enzymes, increasing concentrations of the inhibitors were added to constant amounts of the enzymes and residual activity was monitored using the substrate, Leu-Gly-Arg-NHMec. No inhibition of enzyme activity was seen for either serpin with any of the enzymes (data not shown).
